# Modulation of the EMT/MET Process by E-Cadherin in Airway Epithelia Stress Injury

**DOI:** 10.3390/biom11050669

**Published:** 2021-04-30

**Authors:** Li Han, Huaiqing Luo, Wenjie Huang, Jiang Zhang, Di Wu, Jinmei Wang, Jiao Pi, Chi Liu, Xiangping Qu, Huijun Liu, Xiaoqun Qin, Yang Xiang

**Affiliations:** 1 Department of Physiology, School of Basic Medical Science, Central South University, Changsha 410007, China; hanlidemeng@126.com (L.H.); a17377552242@163.com (W.H.); liema123@sina.cn (J.Z.); 186511007@csu.edu.cn (D.W.); w1321764971@163.com (J.W.); 186511006@csu.edu.cn (J.P.); liuchi7669@gmail.com (C.L.); quxiangping@csu.edu.cn (X.Q.); liuhuijun@csu.edu.cn (H.L.); 2 Department of Physiology, School of Basic Medicine, Changsha Medical University, Changsha 410219, China; luohuaiqing@163.com

**Keywords:** E-cadherin, injury repair, epithelial-mesenchymal transformation, mesenchymal–epithelial transformation, TGFβ1, β-catenin

## Abstract

Persistent injury and the following improper repair in bronchial epithelial cells are involved in the pathogenesis of airway inflammation and airway remodeling of asthma. E-cadherin (ECAD) has been shown to be involved in airway epithelium injury repair, but its underlying mechanisms to this process is poorly understood. Here, we describe a previously undetected function of ECAD in regulating the balance of EMT and MET during injury repair. Injury in mice and human bronchial epithelial cells (HBECs) was induced by successive ozone stress for 4 days at 30 min per day. ECAD overexpression in HBECs was induced by stable transfection. EMT features, transforming growth factor beta1 (TGF-β1) secretion, transcriptional repressor Snail expression, and β-catenin expression were assayed. Ozone exposure and then removal successfully induced airway epithelium injury repair during which EMT and MET occurred. The levels of TGF-β1 secretion and Snail expression increased in EMT process and decreased in MET process. While ECAD overexpression repressed EMT features; enhanced MET features; and decreased TGF-β1 secretion, Snail mRNA level, and β-catenin protein expression. Moreover, activating β-catenin blocked the effects of ECAD on EMT, MET and TGF-β1 signaling. Our results demonstrate that ECAD regulates the balance between EMT and MET, by preventing β-catenin to inhibit TGFβ1 and its target genes, and finally facilitates airway epithelia repair.

## 1. Introduction

Bronchial epithelial cells are the first barrier to various allergens and environmental hazards. Persistent airway epithelial damage and the following improper repair in bronchial epithelial cells are involved in the pathogenesis of airway inflammation and airway remodeling, which are considered as the pivotal factors most closely associated with the incidence and prognosis of asthma [1]. Therefore, clarifying the events of injury repair in bronchial epithelial cells in response to harmful stresses may shed light into developing therapies for asthma.

Injured epithelial cells generally undergo EMT to lose their junctions and apical-basal polarity and reorganize their cytoskeleton; this increases the motility and deformability of individual cells to promote cell migration and invasion, which facilitate epithelial repair, whereas hyperactive EMT has been previously reported to contribute to the pathogenesis of airway inflammation and airway remodeling. In some animal models of lung injury, EMT appears transiently [2,3], no expected fibrosis or remodeling occurred. This suggests that upon most occasions, EMT is self-limiting or reversible, which means there may be a reverse process-mesenchymal-epithelial transition (MET) [4]. In fact, some previous studies have reported that EMT and MET can regulate embryonic stem cell differentiation, induce pluripotency, and cancer stem cell behavior [5,6,7]. Therefore, we speculate that EMT in epithelial cells is a protective response to injury; when proliferation and migration are completed, cells will return to epithelial phenotype (MET) and finally complete repair. So, if EMT cannot be timely limited or MET cannot occur, the balance of injury repair will be disrupted and finally results in fibrosis or reconstruction. Although the factors involved in regulating EMT and MET are still incompletely defined, some clues point to the dynamic changes of epithelial adhesion contacts [8].

Adhesion protein E-cadherin (ECAD) mediates homologous adhesion by repeated extracellular domains to maintain the structural integrity and polarization of epithelia. Its intracellular domains anchor onto the actin cytoskeleton through catenins to form stable connections with adjacent cells and participate in the regulation of signal pathways [9,10]. A number of studies have shown that ECAD in bronchial epithelial cells participates in injury repair and its downregulation is a crucial factor in the progression of asthma [11,12]. ECAD loss, an important phenotypic change in EMT, causes cells to dissociate from their neighbors and cell polarity loss which, in turn, results in the activation of cell signaling pathways to regulate mesenchymal transition [13,14]. In contrast, increased expression of ECAD inhibits tumor cell transformation and invasion in an adhesion-independent manner [15]. Additionally, ECAD can induce MET in mouse S180 sarcoma cells and MIAPaCa-2 pancreatic tumor cell lines with upregulation of α- and β-catenin mRNA expression [16]. However, in non-tumor cells, the effect of ECAD on EMT and MET is rarely studied. A relevant study has shown [14] that ECAD inhibits the activation of myofibroblast precursor hepatic stellate cells (HSC) by suppressing TGFβ1/Smad signaling. Therefore, we hypothesized that ECAD may modulate the balance of EMT and MET in airway epithelial cells to promote repair.

To address this hypothesis, we utilized an ozone-stressed injury repair model to identify EMT/MET phenotypic features and ECAD expression changes. Along with the in vivo experiments, we also used a stably transfected HBEC lines in vitro model to validate the role of ECAD in regulating the balance between EMT and MET and unravel the underlying mechanisms during the process of ozone-induced injury repair.

## 2. Materials and Methods

### 2.1. Animal Model of Injury Repair

BALB/c mice (6–8 weeks) weighing 15–20 g were obtained from the Laboratory Animal Centre of Central South University, China, and housed in a specific pathogen-free conditions. All animal experiments complied with the ARRIVE guidelines and were carried out according to the National Institutes of Health Guide for the Care and Use of Laboratory Animals approved by the Central South University at XiangYa Animal Care and Use Committee. BALB/c mice were randomly divided into six experimental groups: (1) Control group, (2) Ozone group, (3) Repair for 1 day (Repair 1d), (4) Repair for 2 days (Repair 2d), (5) Repair for 3 days (Repair 3d), and (6) Repair for 4 days (Repair 4d). To construct the model of injury repair, mice were exposed to ozone at 2.0 ppm for 30 min per day for 4 days and then removed from ozone atmosphere for healing. Ozone was generated by an ozone generator (Model LT-100, Litian, Beijing, China). In addition, the ozone concentration in the chamber was continuously monitored with an ozone meter (EG-2001; Ebara Jitsugyo, Tokyo, Japan). During exposure, the control mice were always treated with fresh air [17].

### 2.2. Measurement of Airway Function

For all groups, airway responsiveness expressed by pulmonary resistance (R_L_) was assessed 2 h after the end of exposure. Briefly, mice were anesthetized by i.p. injection of ketamine/xylazine cocktail (50 and 10 mg/kg, respectively), then placed in a whole-body chamber, and basal readings were obtained. After baseline determination of R_L_, mice were challenged with methacholine (3.12 mg/mL in sterile Saline; Sigma-Aldrich, St. Louis, MO, USA). R_L_ data were measured by direct plethysmography determined by multiple linear regressions from transpulmonary pressure and airflow (Biosystems XA; Buxco Electronics, Harvard Bioscience, lnc., Holliston, MA, USA). For each animal, we calculated the average of the 3 highest measurements of R_L_ and used these values/baseline values to obtain dose–response curves.

### 2.3. Histology, H&E, and Immunochemistry

Lower right lungs were fixed in 4% paraformaldehyde for 24 h, and then, they were processed for paraffin embedding. Hematoxylin and eosin (H&E) staining for structured and inflammatory observation was conducted on 5 μm sections according to previously published procedures [18]. Immunohistochemistry (IHC) analyses were performed on mouse lung paraffin sections by employing anti-E-Cadherin (SAB4503751 1:200; Sigma-Aldrich, St. Louis, MO, USA), anti-α-smooth muscle actin (α-SMA; SAB5500002 1:200; Sigma-Aldrich). Zeiss Axio Scope. A1 or Zeiss Discovery. V8 Stereo microscopes (Carl Zeiss MicroImaging GmbH, Göttingen, Germany) was used and integrated with an Axio-Cam ICc3 camera (Spectra Service, Ontario, NY). Images were acquired by AxioVision Rel. 4.7 software from Zeiss. To avoid the impact of altered airways after ozone treatment, we detected ECAD and α-SMA expression based on the same epithelial cell number from different groups.

The remained right lungs were harvested (ice operation), and placed into a labeled tube frozen with liquid nitrogen and stored at −70 °C for real-time PCR.

### 2.4. Cell Culture and Model of Injury Repair

Human bronchial epithelial cell, 16HBE14o−, was generously gifted by Dr. Dieter C Gruenert, University of California San Francisco [19]. Overexpression of E-cadherin in HBEC lines was induced by stable transfection. These cells were cultivated in high-glucose Dulbecco’s modified Eagle’s medium (DMEM) supplemented with 10% fetal bovine serum (FBS), 100 U/mL penicillin, and 100 U/mL streptomycin in 5% CO_2_ at 37 °C. In addition, the medium for stably transfected HBEC lines was supplemented with 200 μg/mL G-418 sulfate. For treatment of cells, the corresponding serum-containing media were replaced with DMEM containing 3% FBS.

Cells were seeded into six-well tissue plate at a density of 1–2 × 10^5^ cells per well and grown to 50–60% confluence for experiment. Cells were treated with ozone (1.5 ppm) for 30 min per day for 4 days under culture conditions, then allowed to repair for 4 days with filtered air. Ozone was produced by a commercial ozonator (Model LT-100, Litian, Beijing, China).

To evaluate the effect of β-catenin on ECAD-inhibiting TGFβ1-Snail signaling, we added the medium with LiCl, a small-molecule compound that specifically activates β-catenin signaling, as previously described [20]. Specifically, ECAD-overexpressed cells lines were cultured in nutrient solution with LiCl (ECAD + LiCl group) or without it (ECAD group) until the cells were harvested.

### 2.5. Overexpression Plasmid Synthesis and Transfection

An effective E-cadherin RNA (forward: 5′-ACGGGCCCTCTAGACTCGAGATGGGCCCTTGGAGCCGCAG-3′, reverse: 5′-AGTCACTTAAGCTTGGTACCGAGTCGTCCTCGCCGCCTCCGTACATG-3′) and GV112, a nonsense RNA (forward: 5′-CCUCUUUGACUAUUACACCAGGCUU-3′, reverse: 5′-AAGCCUGGUGUAAUAGUCAAAGAGG-3′), were synthesized by Genechem Co., Ltd. (Shanghai, China). Transfections were conducted with Lipofectamine 3000 (Invitrogen; Thermo Fisher Scientific, Inc., Waltham, MA, USA) according to the manufacturer’s instructions. Stably transfected HBEC lines were obtained after selection by G418 at 500 ug/mL and verified by real-time PCR and Western blotting analysis.

### 2.6. Total RNA Extraction and Real-Time PCR

Total RNA was extracted from lung tissue regions or HBECs with TRIzol (Invitrogen, Carlsbad, CA, USA). Briefly, 1 μg of mRNA was reverse-transcribed into cDNA for PCR amplification with PrimeScript™ RT reagent Kit (Takara Bio, Tokyo, Japan) according to manufacturer’s instruction. Amplification was conducted in a 20 μL of diluted cDNA with the SYBR Green reagents (Takara Bio, Japan) using CFX96 Touch^TM^ Real-Time PCR machine (Bio-Rad, Hercules, CA, USA). The steps included 40 cycles of 95 °C for 30 s, 95 °C for 5 s, 60 °C for 30 s. Each sample was run in triplicate. Target gene expression was normalized to the expression of a cellular housekeeping gene, beta actin (β-actin), and calculated using the 2^−ΔΔCT^ method. The primer sets (Sangon, Shanghai, China) used for real-time PCR are listed in Table 1.

### 2.7. Western Blotting

Western blotting analysis was performed as described previously [21]. Briefly, cells were lysed in RIPA lysis buffer containing 1% phenylmethanesulfonyl fluoride (PMSF) (Sigma-Aldrich). In addition, nuclear extracts were prepared with nuclear and cytoplasmic extraction reagent kit (Thermo Fisher Scientific) in accordance with the manufacturer’s instructions for the detection of β-catenin protein expression. Proteins in supernatants were separated by 8–10% SDS-PAGE and electrotransferred to polyvinylidene fluoride (PVDF) membranes (Millipore, Billerica, MA, USA). The membranes were incubated with primary antibodies as follows Anti-E-cadherin (#3195, 1:1000; Cell Signaling Technology, Boston, MA, USA), anti-Cytokeratin 19 (ab15463, 1:1000; Abcam, Cambridge, UK), anti-α-SMA (ab247668, 1:1000, Abcam), anti-β-catenin (ab32572, 1:1000; Abcam), Anti-β-actin (A1978, 1:10,000; Sigma-Aldrich) and subsequently reacted with relative secondary antibody (Anti-rabbit IgG, #7074 1:10,000; Anti-mouse IgG, #7076 1:10,000, Cell Signaling Technology) prior to visualizing using ECL reagents (Millipore). Film density was measured using ImageJ densitometry software and normalized against β-actin.

### 2.8. Immunofluorescence

Cells from different stage of injury repair were fixed with 4% paraformaldehyde for 20 min at room temperature, rinsed three times, and then permeabilized with 0.5% Triton X-100 for 5 min. Cells on slides were stained with phalloidin (Yeasen Bio-technology Co. Ltd., Shanghai, China) for 30 min at room temperature. After washing with PBS, stained samples were incubated with 4′,6-diamidino-2-phenylindole (Sigma-Aldrich) for 2 min. Images were snapshotted by a Zeiss LSM710 confocal microscope (Carl Zeiss).

### 2.9. Enzyme-Linked Immunosorbent Assay (ELISA)

Cell supernatant was used to determine the expression levels of TGFβ1 using ELISA kits according to the manufacturer’s protocol.

### 2.10. Statistical Analysis

All data were performed by SPSS v22.0 (SPSS Inc., Chicago, IL, USA) and presented as mean ± SEM. The *p* value < 0.05 was considered as statistically significant. Statistical differences between two groups were calculated with Student’s *t*-test, whereas differences among multiple groups were analyzed using one-way ANOVA followed by Tukey’s post hoc test.

## 3. Results

### 3.1. ECAD Correlates with EMT/MET and Airway Epithelia Injury Repair in the Ozone-Stressed Mice Model

**Figure 1:**
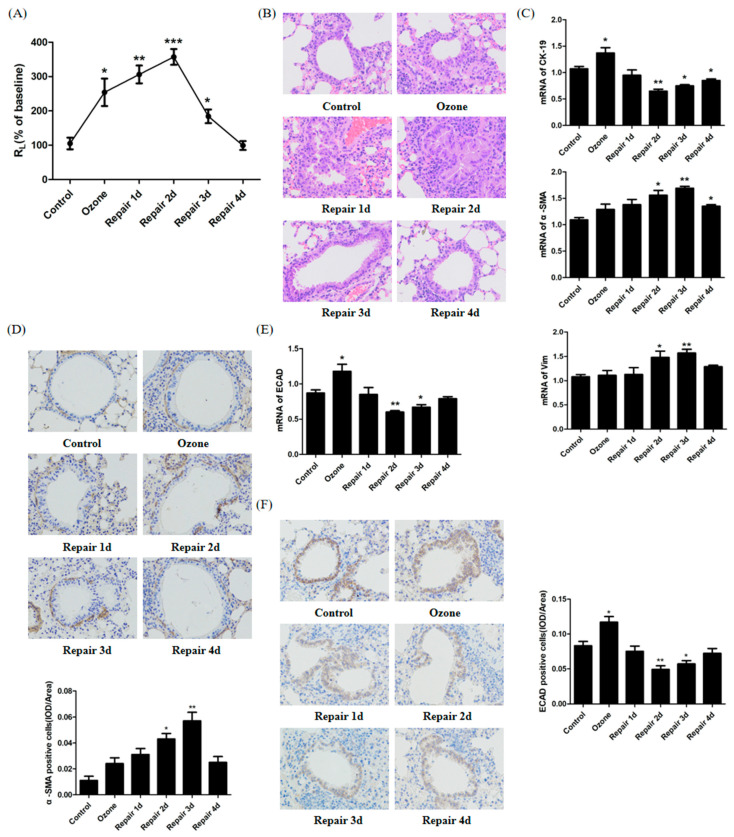
E-cadherin (ECAD) expression and epithelial–mesenchymal transition (EMT)/mesenchymal–epithelial transition (MET) features during airway epithelia injury repair in ozone-stressed mice model. (**A**) Airway hyperresponsiveness (AHR). Changes in pulmonary resistance (R_L_) induced by inhaled aerosolized methacholine were assessed. N = 6~8 in each group. (**B**) H&E staining of lung tissues from different stages of injury repair. All images were obtained at 200× magnification. N = 3 in each group. (**C**) mRNA expressions of CK-19, α-SMA, and Vim from different stages of injury repair. N = 3 in each group. (**D**) Immunoreactivity of α-SMA from different stages of injury repair. Images of each group were obtained at 200× magnification. N = 3 in each group. (**E**) mRNA expressions of ECAD in airway epithelia from different stages of injury repair. N = 3 in each group. (**F**) Immunoreactivity of ECAD in airway epithelia of mice from different stages of injury repair. N = 3 in each group. All data are presented as mean ± SEM of 3 independent experiments. * *p* < 0.05, ** *p* < 0.01, *** *p* < 0.001 versus control group.

To confirm the occurrence of airway epithelia injury repair, pulmonary resistance (R*_L_*) and a histological analysis were performed. The R*_L_* measurements showed that significant AHR was induced after ozone stress and further enhanced until 2 days after repair. AHR was relieved significantly from 3 days after repair. By 4 days after repair, RL returned to the normal levels (Figure 1A). In addition, hematoxylin and eosin (H&E) staining of lung tissues from ozone stress mice demonstrated inflammatory cell peribronchial infiltrates, visible epithelial fractures, epithelial cell shedding, mild bronchiole smooth muscle hypertrophy, and airway wall thickness. The above symptoms further aggravated until 2 days after repair, but began to relieve 3 days after repair (Figure 1B).

To define whether EMT/MET occurred during the injury repair, we examined the expression of epithelial marker CK-19 and mesenchymal markers α-SMA and vimentin (Vim). The results from the real-time PCR showed that the mRNA of CK-19 stressfully increased after ozone exposure, then decreased gradually to the lowest level 2 days after repair, whereas α-SMA and Vim expression increased gradually until 3 days after repair. These results indicated that EMT features were presented in injury repair. At day 4 of repair, the expression of CK-19 increased with a reciprocal decrease in α-SMA and Vim, and this demonstrated that EMT was weakened and began to shift to the epithelial phenotype, namely, MET occurrence (Figure 1C). α-SMA protein expression was detected by immunohistochemistry (IHC). Consistent with the real-time RT-PCR results, α-SMA protein expression increased after ozone stress (Figure 1D). α-SMA protein expression was further increased until 3 days after repair but began to decrease 4 days after repair. To understand the relationship between ECAD and EMT/MET as well as airway epithelia injury repair, time-dependent changes in ECAD expression were monitored in airway epithelium cells as in Figure 1E,F. The results of mRNA gene expression showed that ECAD, a most important epithelial marker, decreased in the EMT process but increased in the MET process. In terms of injury repair, ECAD expression level decreased gradually in the initial stage of injury repair but increased with the completion of repair (Figure 1E). To verify the real-time PCR results, we confirmed ECAD protein expression in airway epithelium in mice using immunocytochemistry, as shown in Figure 1F.

### 3.2. EMT/MET Features in Bronchial Epithelial Cells in Ozone-Induced Injury Repair

**Figure 2:**
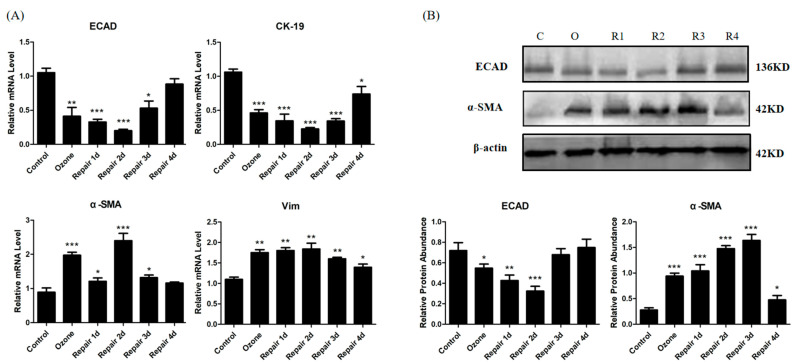
EMT/MET molecular phenotype in human bronchial epithelial cells (HBECs) in ozone-induced injury repair. (**A**) mRNA expressions of ECAD, CK-19, α-SMA, and Vim from different stages of injury repair in HBECs. (**B**) Representative Western blot analysis of ECAD and α-SMA expression in HBECs during ozone-induced injury repair and quantification relative to β-actin in different stages of HBECs. C: Control; O: Ozone; R1: Repair 1d; R2: Repair 2d; R3: Repair 3d; R4: Repair 4d. All data are presented as mean ± SEM of 3 independent experiments. * *p* < 0.05, ** *p* < 0.01, *** *p* < 0.001 versus control group, N = 3 in each group.

As shown in Figure 2A, ozone treatment for 4 consecutive days induced the molecular EMT phenotype in HBECs, consisting of down-regulation of the epithelial marker CK-19 and up-regulation of the mesenchymal markers α-SMA and Vim. Moreover, CK-19 downregulated until 2 days after repair, whereas α-SMA and Vim upregulated; this demonstrated that EMT process was further developed. 3 days after repair, CK-19 expression began to upregulate, while α-SMA and Vim downregulate, indicating molecular MET phenotype emerged. However, the expression levels of the epithelial and mesenchymal markers did not recover to the normal level at day 4 of repair.

To verify the real-time PCR results, Western blot was used to confirm ECAD and α-SMA protein expression in HBECs. As shown in Figure 2B, ECAD protein expression remarkably reduced after ozone stress and gradually descended to the lowest level at day 2 of repair, while α-SMA protein expression increased significantly. There was an increase of ECAD expression from the third day of repair. Although α-SMA protein expression decreased on day 4 of repair, it remained higher than that in normal culture group.

### 3.3. ECAD Overexpression Represses EMT Features and Promotes MET Features in Ozone-Stressed HBECs

**Figure 3:**
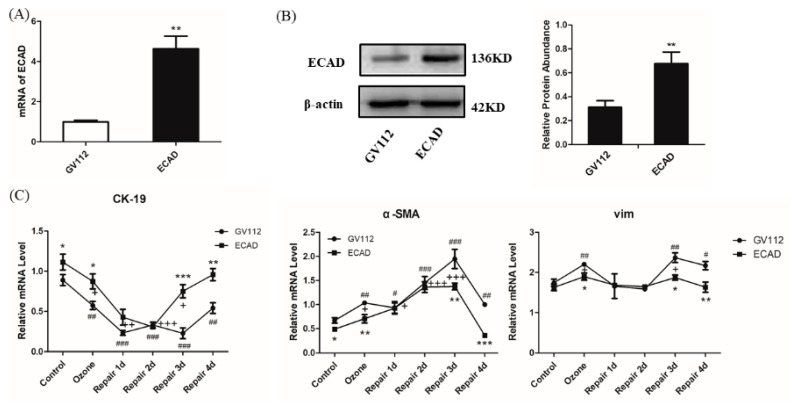

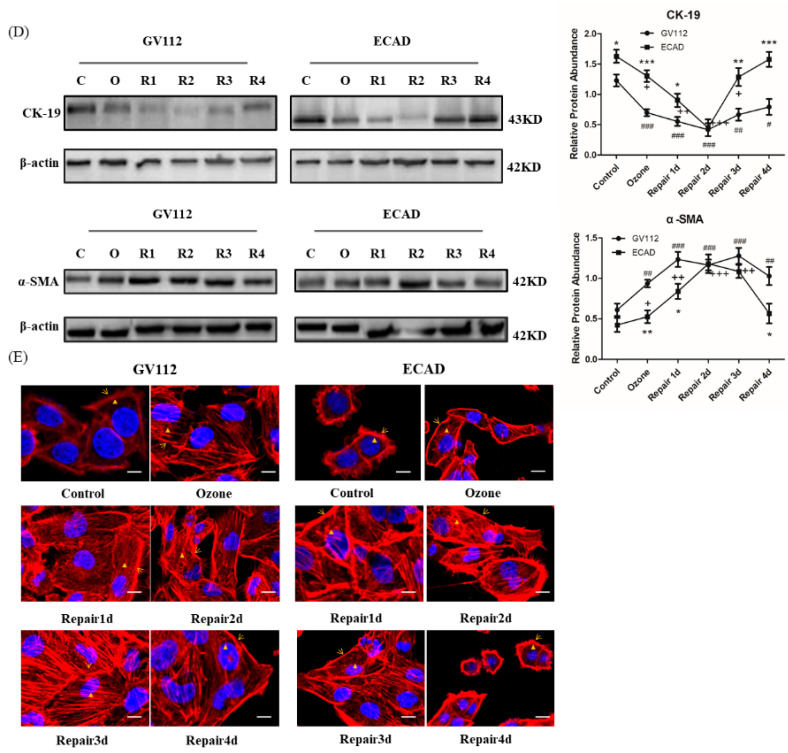
ECAD represses EMT and facilitates MET in HBECs in ozone-induced injury repair. (**A**,**B**) ECAD mRNA expression and protein expression following ECAD-stable transfection were detected by real-time PCR and Western blot analysis. (**C**) Real-time PCR analysis showing EMT/MET markers’ (CK-19, α-SMA, and Vim) expression in ECAD-overexpressed HBECs in ozone-induced injury repair. (**D**) Representative Western blot analysis of CK-19 and α-SMA expression in ECAD-overexpressed HBECs in ozone-induced injury repair and quantification relative to β-actin in different stages of HBECs. C: Control; O: Ozone; R1: Repair 1d; R2: Repair 2d; R3: Repair 3d; R4: Repair 4d. (**E**) Fluorescence images (blue, nucleus; red, F-actin; scale bar, 20 μm) of HBECs stably transfected with ECAD in ozone-induced injury repair. All data are presented as mean ± SEM of 3 independent experiments. ^#^ *p* < 0.05, ^##^ *p* < 0.01, ^###^ *p* < 0.001 versus control group in GV112-transfected HBECs; ^+^ *p* < 0.05, ^++^ *p* < 0.01, ^+++^ *p* < 0.001 versus control group in ECAD-transfected HBECs; * *p* < 0.05, ** *p* < 0.01, *** *p* < 0.001 versus GV112 group from the same stage, N = 3 in each group.

To better understand the relationship between ECAD and EMT/MET during injury repair, we overexpressed ECAD in HBECs (Figure 3A,B) and explored the effect of ECAD on EMT/MET marker expression. As shown in Figure 3C, epithelial marker CK-19 was gradually down-regulated until 2 days after repair but mesenchymal markers α-SMA and Vim were up-regulated. From 3 days after repair, CK-19 expression began to increase and α-SMA and Vim decrease 4 days after repair. These findings suggested the presence of molecular EMT/MET phenotype in GV112-transfected HBECs (control group) during the process of injury repair. However, at day 4 of repair, CK-19, α-SMA, and Vim did not restore completely. Injured epithelial cells generally undergo EMT to acquire the ability of migration and deformation to facilitate epithelial repair. Ozone exposure also induced molecular EMT phenotype in ECAD-overexpressed HBECs, including significantly decrease of CK-19 and increase of α-SMA and Vim. Although ECAD overexpression induced higher expression of CK-19 and lower expression of Vim and α-SMA than control treatment (GV112) at initial stages of repair; but at day 2 of repair, no differences in the expression of EMT markers could be observed. These results indicated that ECAD attenuated rather than preventing EMT. At later stages of repair, there was more increase of CK-19 and more decrease of Vim and α-SMA in ECAD group than those in control group (GV112), and most importantly, at day 4 of repair, above markers in ECAD group recovered to the normal level suggesting the occurrence of MET and the return to epithelial phenotype, as well as the completion of repair. CK-19 and α-SMA protein expression in HBECs detected by Western blot verified the real-time PCR results (Figure 3D).

Another important event in the EMT/MET process is cytoskeletal reorganization. So, we also assessed the F-actin fibers using phalloidin staining as shown in Figure 3E. The results revealed that peripheral F-actin fibers were continuous, a distribution typical of epithelial cells, in the control group of GV112-transfected HBECs. After ozone exposure for 4 days, the continuity of the peripheral F-actin fibers was disrupted and thick F-actin fibers were significantly increased throughout the cell body (arrows and triangles), indicating a motile, mesenchymal-like phenotype. These mesenchymal-like cytoskeletal changes further developed until 3 days after repair and weakened at day 4 of repair. Compared with the GV112 group, ECAD overexpression alone enhanced the peripheral distribution of F-actin fibers. After 4 days of continuous ozone stress, the F-actin fibers in ECAD-overexpressed HBECs also showed mesenchymal-like cytoskeletal changes. However, these changes were significantly lower than those in GV112-transfected HBECs and began to weaken from 3 days after repair. At day 4 of repair, F-actin fibers re-localized and reorganized to the cortical region of the HBECs.

### 3.4. The Effects of ECAD on EMT/MET Is Mediated by TGFβ1/Snail Signaling

**Figure 4:**
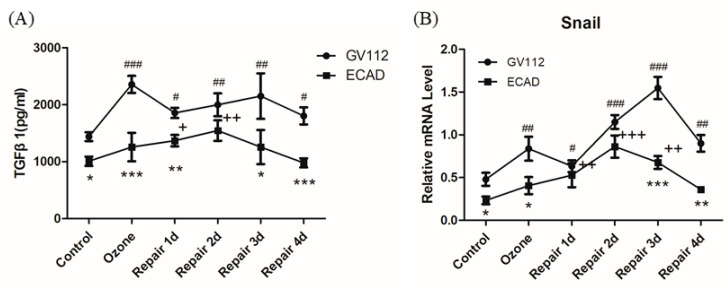
ECAD modulates EMT/MET by inhibiting transforming growth factor β1(TGFβ1)-Snail signaling. (**A**) ELISA analysis of TGFβ1 secretion in HBECs stably transfected by ECAD in ozone-induced injury repair. (**B**) mRNA expression of Snail in HBECs following ECAD stable transfection were detected by real-time PCR in ozone-induced injury repair. All data are presented as mean ± SEM of 3 independent experiments. ^#^ *p* < 0.05, ^##^ *p* < 0.01, ^###^ *p* < 0.001 versus control group in GV112-transfected HBECs; ^+^ *p* < 0.05, ^++^ *p* < 0.01, ^+++^ *p* < 0.001 versus control group in ECAD-transfected HBECs; * *p* < 0.05, ** *p* < 0.01, *** *p* < 0.001 versus GV112 group from the same stage, N = 3 in each group.

TGFβ1 signaling is involved in several biological processes, including EMT [22]. Therefore, the effect of ECAD overexpression on TGFβ1 was monitored. As shown in Figure 4A, ozone exposure induced significant increase of TGFβ1 secretion. In addition, TGFβ1 release increased until 3 days after repair but began to decrease at day 4 of repair. The dynamic changes of TGFβ1 secretion were similar with mesenchymal markers which revealed its regulating effect on ETM/MET. When compared with GV112-transfected HBECs, ECAD elicited a significant decrease in TGFβ1 secretion through the EMT/MET process. It has been reported that TGFβ1 promotes EMT through transcriptional repressors, such as the zinc finger-containing proteins Snail. As we expected, a similar change was observed in the expression of snail (Figure 4B).

### 3.5. Role of β-catenin in ECAD Inhibition Effect on TGFβ1/Snail Signaling

**Figure 5:**
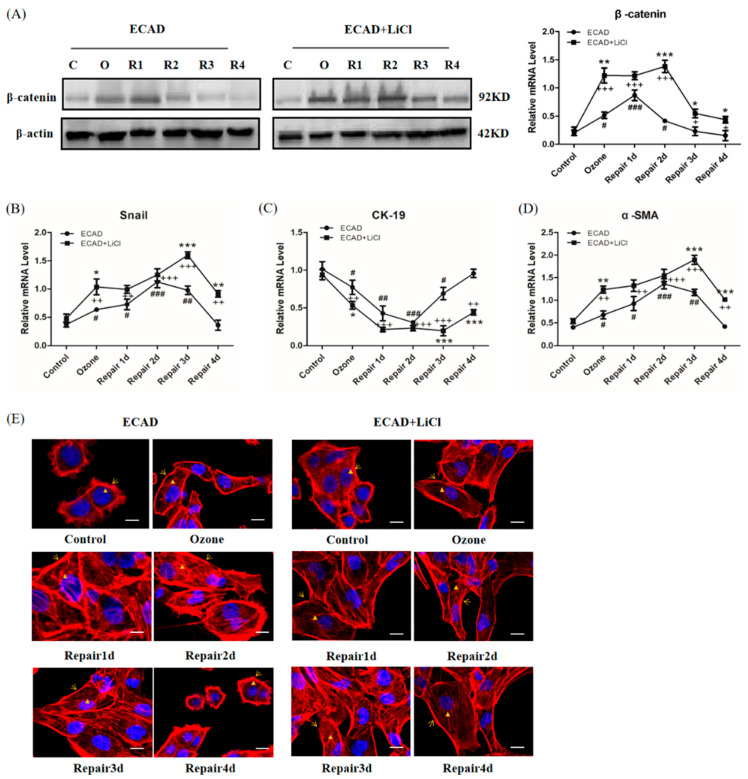
Association of β-catenin with ECAD for the repression of Snail activity. (**A**) Representative Western blot analysis of β-catenin expression in ECAD-overexpressed HBECs treated with LiCl in ozone-induced injury repair and quantification relative to β-actin in different stages of HBECs. C: Control; O: Ozone; R1: Repair 1d; R2: Repair 2d; R3: Repair 3d; R4: Repair 4d. (**B**) mRNA expression of Snail in ECAD-overexpressed HBECs treated with LiCl during the ozone-induced injury repair. (**C**) mRNA expressions of CK-19 in ECAD-overexpressed HBECs treated with LiCl. (**D**) mRNA expressions of α-SMA in ECAD-overexpressed HBECs treated with LiCl. (**E**) Fluorescence images (blue, nucleus; red, F-actin; scale bar, 20 μm) of ECAD-overexpressed HBECs treated with LiCl in ozone-induced injury repair. All data are presented as mean ± SEM of 3 independent experiments. ^#^ *p* < 0.05, ^##^ *p* < 0.01, ^###^ *p* < 0.001 versus control group in ECAD group; ^+^ *p* < 0.05, ^++^ *p* < 0.01, ^+++^ *p* < 0.001 versus control group in ECAD + LiCl group; * *p* < 0.05, ** *p* < 0.01, *** *p* < 0.001 ECAD + LiCl versus ECAD group from the same stage, N = 3 in each group.

To verify the regulatory role of β-catenin in Snail activation, the expression level of Snail and Snail-dependent gene (CK-19, α-SMA) were monitored in cells treated with LiCl (a β-catenin activator). As shown in Figure 5A,B, the treatment of ECAD overexpression cells with LiCl induced an increase in β-catenin and Snail expression during the EMT/MET process, and enhanced the effect of Snail on CK-19 (Figure 5C) and α-SMA (Figure 5D). Furthermore, F-actin fibers were also detected, showing that LiCl increased F-actin fibers throughout the cell body (Figure 5E).

**Figure 6:**
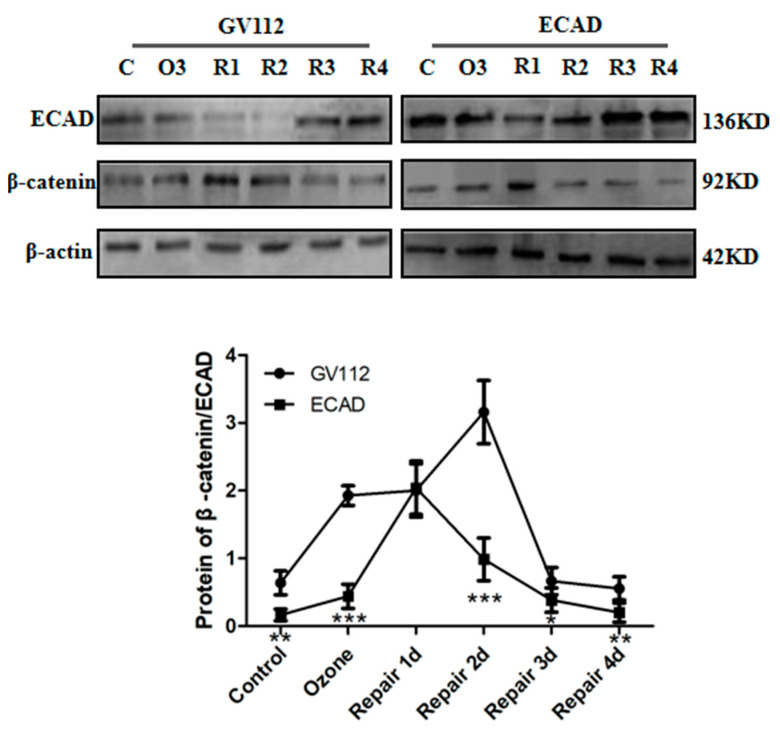
ECAD inhibits β-catenin expression. Representative Western blot analysis of ECAD and β-catenin expression in ECAD-overexpressed HBECs during ozone-induced injury repair and quantification relative to β-actin in different stages of HBECs. C: Control; O: Ozone; R1: Repair 1d; R2: Repair 2d; R3: Repair 3d; R4: Repair 4d. All data are presented as mean ± SEM of 3 independent experiments. * *p* < 0.05, ** *p* < 0.01, *** *p* < 0.001 versus GV112 group from the same stage, N = 3 in each group.

In addition, the effect of ECAD on β-catenin was detected by Western blot. The results revealed that ECAD significantly decreased the β-catenin expression during the EMT/MET process (Figure 6).

## 4. Discussion

In this study, we examined the effects and underlying mechanisms of ECAD on ozone-induced injury repair in airway epithelium. Our findings verified that EMT/MET occurred during the injury repair in HBECs induced by consecutive ozone stress. The EMT/MET features included marker expression, cytoskeleton reorganization, mRNA expression of the transcription factors Snail, and the release of TGF-β1. ECAD regulates the balance between EMT and MET, by preventing β-catenin to inhibit TGFβ1/Snail signal, and finally facilitates airway epithelia repair. This study helps to provide a novel insight for the beneficial role of ECAD in airway epithelium repair and reveal the possible mechanisms underlying the effect of ECAD on bronchial asthma.

Ozone, a ubiquitous air pollutant, can cause airway epithelial cell damage [23] and asthmatic symptoms such as airway inflammation and AHR [24]. Our results showed that ozone stress (1.5 ppm, 30 min/day, for 4 consecutive days) induced significant AHR, airway inflammation and structural disruption and shedding of epithelial cells, which relieved from 3 days after repair; these indicated that injury repair model was successfully constructed (Figure 1A,B).

Converging lines of evidence have identified EMT as an essential component of physiologic tissue repair, during which epithelial cells lose their intercellular adhesions and migrate across the wound to restore the epidermal barrier [25,26]. To determine if EMT/MET occur during the process of injury repair in airway epithelium, we examined the changes of EMT/MET features in mice (Figure 1C–F) and HBECs (Figure 2 and Figure 3E). Cells in the EMT process alter their gene expression program, including a decrease of ECAD expression and an increase of the mesenchymal marker α-SMA [3,27]. In addition, the genes Vim and CK-19 have also been found to contribute to rearrangement of the cytoskeletal architecture in EMT [5]. In the present study, we found EMT phenotype, including CK-19 decrease and α-SMA and Vim increase and a mesenchymal-like reorganization of F-actin fibers, in the early stage of injury repair. While in the late stage (from 3 days after repair), the molecular EMT phenotype began to reversion and an epithelial-like reorganization of F-actin fibers appeared. These results verify our speculation. In addition, a difference between experiments in vivo and in vitro was found: epithelial marker CK-19 was stressfully upregulated after ozone exposure in vivo. This may play a protective role for airway epithelial injury. These findings imply that the balance of EMT and MET may be the key event for normal airway epithelia repair. Previous studies found that the airway epithelia of asthma patients are more likely to activate an abnormal interstitial repair process in response to daily injury due to defective epithelial repair and dysregulation of structural and functional homeostasis [28,29], which support our hypothesis to some extent.

ECAD, a most important epithelial marker, has been found to have a dynamic change in EMT/MET during the process of injury repair in airway epithelia (Figure 1E,F and Figure 2). Loss of ECAD has been shown to disrupt cell adhesion, resulting in enhanced cellular motility and EMT in cancer cells [30]. Conversely, forced expression of ECAD shows an inhibiting effect on cell transformation and tumor cell invasion [31]. Although ECAD had been recognized as a driver of EMT/MET in cancer progression, rare information was available about the role of ECAD in EMT/MET in normal tissue and cells in response to injury. One previous study showed that ECAD caused mesenchymal–epithelial transition in rat kidney fibroblast NRK 49f cells and prevented EMT in rat kidney tubular epithelial cells NRK52e [32]. However, no studies have established the role of ECAD in the transformation process of epithelial cells during injury repair. To verify the regulated effects of ECAD on EMT/MET during injury repair, a stably transfected HBECs was constructed and stressed by ozone for 4 consecutive days. After ozone stress, forced ECAD expression induced higher epithelial marker (CK-19) expression and a more normal structure of F-actin fibers in airway epithelial cells than control treatment. These observations imply that ECAD-overexpressed HBECs have stronger resistance against damage. Furthermore, our data showed that EMT and MET features also emerged in ECAD overexpression HBECs. During the process of repair, forced ECAD expression induced higher epithelial marker (CK-19) expression, lower mesenchymal markers (α-SMA and Vim) expression, and an earlier epithelial-like reorganization of F-actin fibers in airway epithelial cells, when compared with control (GV112) group. More importantly, ECAD made the EMT markers and F-actin fibers distribution approach to the normal level at day 4 of repair (Figure 3). EMT- and MET-like events are coordinated during this process. Taken together, these results demonstrate that ECAD may be an important modulator to the balance of EMT and MET by suppressing EMT hyperactivity and enhancing its reverse (MET) to promote airway epithelial repair.

Several lines of evidence indicate that exogenous TGFβ1 promotes epithelial wound repair by inducing EMT [33], while excessive production of TGFβ1 results in fibrosis. As we expected, TGFβ1 secretion increased in the process of ozone-induced EMT and decreased in MET. Similarly, a decrease in TGFβ1 release has been found during the MET process of tumors [34]. TGFβ1 represses the expression of ECAD and promotes the following temporal sequence: disassembly of cell junctions, loss of epithelial polarity, cytoskeletal reorganization, and cell–matrix adhesion remodeling. Although the disintegration and disassembly of cell-cell junctions by TGFβ1 is the well-known process, information about whether ECAD has an inhibitory effect on TGFβ1 expression in airway epithelial cells was not available. Our results demonstrate that ECAD prevents TGFβ1 release through the EMT/MET process (Figure 4A).

Among several transcription factors, Snail1 seems to be a master regulator of EMT [5,35,36]. It can be regulated by TGF-β1 merged by downstream signals and acts partly by repressing expression of ECAD and induction of Vim expression [37]. Repression of Snail1 expression is usually sufficient to induce ECAD expression and the cells acquire an epithelial phenotype through initiation of mesenchymal–epithelial transition (MET) [35]. In consistent with TGF-β1, ECAD also represses snail genes through the EMT/MET process (Figure 4B). These results suggested that ECAD is involved in the regulation of TGF-β signal, orchestrates a dynamic balance of epithelial–mesenchymal signals that induce EMT- and MET-like differentiation processes. This paradigm may be relevant not only for airway epithelium injury repair but also other physiological and pathological process that involve EMT.

β-catenin stabilizes ECAD to maintain the structure and functional homeostasis of epithelial cells. Altered localization and increased expression of β-catenin in the cytoplasm are associated with embryo development, tissue fibrosis, and cancer progression [32]. The important finding of our study is the identification of β-catenin as a crucial molecule of ECAD in inhibiting TGFβ1 signaling during the EMT/MET process (Figure 5 and Figure 6). This is supported by the following observations: ECAD inhibits β-catenin expression, and β-catenin activator LiCl blocks ECAD’s inhibition of Snail and its target gene expressions as well as mesenchymal-like reorganization of F-actin fibers during the process of injury repair in HBECs. Therefore, the signaling pathway mediated by β-catenin bound to ECAD may be responsible for TGFβ1 repression in cells of the epithelial type, which still needs further studies.

## 5. Conclusions

In conclusion, ECAD inhibits EMT hyperactivity and promotes MET, by regulating β-catenin to suppress TGFβ1 signal, and finally facilitates airway epithelia repair. Our results show a reciprocal correlation between ECAD expression and EMT/MET in airway epithelia. To the best of our knowledge, it is the first time to provide identification of ECAD effect on regulating EMT/MET in airway epithelium during the injury repair. This provides a new avenue to precisely understand the pathogenesis of bronchial asthma and offer insights to develop therapies for it.

## Figures and Tables

**Table 1:** Real-time PCR primers used in this study.

Target/Control Gene	Primer Sequences
Mouse ECAD	Forward 5′-ACCGGAAGTGACTCGAAATGATGT-3′
	Reverse 5′-CTTCAGAACCACTGCCCTCGTAAT-3′
Mouse CK-19	Forward 5′-GGTTCAGTACGCATTGGGTCA-3′
	Reverse 5′-CGGAGGACGAGGTCACGAA-3′
Mouse α-SMA	Forward 5′-CCCAGATTATGTTTGAGACC-3′
	Reverse 5′- TCCAGAGTCCAGCACAATAC-3′
Mouse Vim	Forward 5′-AAGCACCCTGCAGTCATTCA-3′
	Reverse 5′- AGGCTTGGAAACGTCCACAT-3′
Mouse β-actin	Forward 5′-TTGCAGCTCCTTCGTTGCC-3′
	Reverse 5′-GACCCATTCCCACCATCACA-3′
Human β-actin	Forward 5′-TTCCAGCCTTCCTTCCTGGG-3′
	Reverse 5′-TTGCGCTCAGGAGGAGCAAT-3′
Human ECAD	Forward 5′-TCCAGGAACCTCTGTGATGGA-3′
	Reverse 5′-ACTCTCTCGGTCCAGCCCA-3′
Human CK-19	Forward 5′-TTTGAGACGGAACAGGCTCT-3′
	Reverse 5′-AGGCTTGGAAACGTCCACAT-3′
Human α-SMA	Forward 5′-GTGTTGCCCCTGAAGAGCAT-3′
	Reverse 5′-GCTGGGACATTGAAAGTCTCA-3′
Human Vim	Forward 5′-TGGACCAGCTAACCAACGAC-3′
	Reverse 5′-GCCAGAGACGCATTGTCAAC-3′

## Data Availability

The data presented in this study are available in insert article.
